# Explainable machine learning for predicting coronary heart disease risk in patients with carotid atherosclerosis: A retrospective study with SHAP and decision curve analysis

**DOI:** 10.1017/cts.2026.10722

**Published:** 2026-03-06

**Authors:** Lei Zhang, Mengke Lyu, Mingyuan Du, Yizhuo Li, Haifeng Yan, Xiaohui Li, Wenshuang Niu, Lizhi Pang

**Affiliations:** 1 Heart Center, The First Affiliated Hospital of Henan University of Chinese Medicine; National Regional (TCM) Cardiovascular Diagnosis and Treatment Center, China; 2 Collaborative Innovation Center of Prevention and Treatment of Major Diseases by Chinese and Western Medicine, China; 3 The First Affiliated Hospital of Henan University of Chinese Medicine, China; 4 The Fifth Clinical Medical College of Henan University of Traditional Chinese Medicine, China; 5 The First Clinical Medical College of Zhengzhou University, China

**Keywords:** Carotid atherosclerosis, coronary heart disease, machine learning, risk prediction, shap

## Abstract

**Background:**

Carotid atherosclerosis is associated with increased coronary heart disease (CHD) risk, yet current risk models lack specificity and interpretability for this population. This study aimed to develop explainable machine learning (ML) models to predict CHD in these patients.

**Methods:**

We retrospectively analyzed 487 patients with carotid atherosclerosis (191 CHD, 296 non-CHD) from January 2022 to July 2025. Thirty-eight variables were collected, including demographic, clinical, and biochemical indicators. LASSO regression identified six key predictors. Seven ML models were trained and evaluated using area under receiver operating characteristic curve (AUC), PRC-AUC, calibration curves, and decision curve analysis (DCA). SHAP was applied to interpret the best-performing model.

**Results:**

Logistic regression model achieved the highest test-set performance (AUC = 0.827; PRC-AUC = 0.752), with strong generalizability and calibration. SHAP analysis identified age and diastolic blood pressure as the most influential features, aligning with model coefficients. DCA demonstrated superior clinical net benefit of the logistic regression model across probability thresholds.

**Conclusion:**

A six-variable logistic model provides accurate and interpretable CHD risk prediction in patients with carotid atherosclerosis. Its transparency and clinical utility support its integration into personalized risk management.

## Introduction

Coronary heart disease (CHD) remains a prominent global cause of mortality and morbidity, responsible for approximately 16% of all deaths worldwide [1]. Its pathogenesis is intricately linked to the development of atherosclerotic plaques and vascular inflammation, with risk factors such as hypertension, dyslipidemia, and diabetes exacerbating disease progression [2]. Importantly, carotid atherosclerosis, characterized by intima-media thickening or plaque formation in the carotid arteries, shares a common pathophysiological basis with CHD, including endothelial dysfunction and lipid accumulation [3,4]. Individuals with carotid atherosclerosis demonstrate a 2–3-fold higher risk of CHD development compared to the general population, underscoring the importance of targeted risk assessment and early intervention in this high-priority cohort [5,6].

CHD risk assessment tools, exemplified by the Framingham Risk Score and SCORE2, predominantly utilize traditional risk factors like age, blood pressure, and smoking status [7]. However, these tools often fail to consider disease-specific markers and intricate interplays among variables. This limitation results in reduced predictive accuracy, particularly in specialized cohorts such as individuals with carotid atherosclerosis. In this context, factors like coagulation function (e.g., thrombin time) and renal function indices (e.g., creatinine) may hold significant predictive value [8]. Moreover, the escalating prevalence of overweight and metabolic syndrome underscores the necessity for comprehensive risk evaluation tools that encompass diverse data dimensions, encompassing anthropometric measurements, biochemical parameters, and clinical history [9,10].

In recent years, machine learning (ML) has emerged as a potent tool for enhancing predictive accuracy in clinical data by capturing intricate nonlinear relationships and high-dimensional interactions [11]. Studies have demonstrated the superior performance of ML models like XGBoost and Random Forest over traditional statistical methods in predicting CHD risk [12]. Nevertheless, a significant obstacle to the widespread adoption of ML in clinical practice is the opaque nature of many sophisticated algorithms, which hinders transparency and trust among healthcare providers [13]. Explainable AI (XAI) methodologies, such as SHAP, mitigate this issue by quantifying the impact of individual features on predictions, thereby improving the interpretability of models [14].

Despite advancements, three primary constraints endure in contemporary research: (1) The majority of ML models for predicting CHD concentrate on the general populace, neglecting sufficient data on individuals with carotid atherosclerosis; (2) Limited research systematically evaluates various ML algorithms” effectiveness, with a focus on clinical applicability such as net benefit assessment through decision curve analysis; (3) The process of feature selection frequently lacks meticulousness, resulting in overfitting or the incorporation of extraneous variables.

This retrospective single-center study aimed to address gaps by developing and validating ML models to predict CHD risk in patients with carotid atherosclerosis. The study utilized 38 candidate variables encompassing demographic, clinical, and biochemical features. Key predictive factors were identified through LASSO regression to enhance model parsimony and generalizability. The study compared the performance of seven ML algorithms (Logistic regression model, Decision Tree, Random Forest, KNN, XGBoost, LightGBM, and Stacking) using various metrics such as area under the receiver operating characteristic curve (AUC), precision-recall, and calibration. Additionally, interpretability was improved through SHAP (SHapley Additive exPlanations) analysis, and clinical utility was evaluated via decision curve analysis to facilitate practical application.

This study aims to enhance the identification of high-risk CHD patients with carotid atherosclerosis by combining explainability and predictive accuracy. The goal is to offer clinicians a dependable tool for tailoring prevention and management strategies to individual patients.

### Study data

This retrospective cross-sectional study examined 487 adult patients aged 18–80 diagnosed with carotid atherosclerosis using ultrasound or computed tomography angiography (CTA) at the First Affiliated Hospital of Henan University of Chinese Medicine between January 1, 2022, and July 20, 2025. Carotid plaque was defined as focal wall thickening exceeding 50% of the surrounding intima-media thickness or carotid intima-media thickness of 1.5 mm or more. Carotid atherosclerosis was characterized by intima-media thickening of 1.0 mm or greater or the presence of atherosclerotic plaque in one or both carotid arteries.

Participants were categorized into two cohorts according to their clinical diagnosis of CHD: the CHD group (*n* = 191) and the non-CHD group (*n* = 296). The diagnosis of CHD followed established criteria outlined in the 2023 ESC Guidelines for Acute Coronary Syndromes Management, encompassing stable angina or acute coronary syndrome. A thorough evaluation was conducted on all participants, encompassing demographic and laboratory assessments. Although ultrasound and CTA were used to confirm the diagnosis of carotid atherosclerosis, detailed imaging-derived metrics (e.g., IMT, plaque characteristics) were not included as predictor variables due to inconsistent availability in the electronic medical records.

Exclusion criteria included congenital heart disease, non-atherosclerotic cardiac conditions such as myocarditis, active malignancy, severe psychiatric disorders, pregnancy or lactation, and participation in other clinical trials within the past 3 months. This retrospective study, approved on April 1, 2024 by the Ethics Committee of The First Affiliated Hospital of Henan University of Chinese Medicine (approval number: 2024HL-159-01), adhered to the principles of the Declaration of Helsinki. Given the retrospective design and use of anonymized clinical data, the requirement for informed consent was waived by the ethics committee. All patient information was anonymized and de-identified before analysis to protect patient privacy in accordance with national health research regulations.

### Data collection and feature variables

Clinical data were obtained from the hospital’s electronic medical records and laboratory information systems and meticulously reviewed by two trained research physicians using a standardized case report form (CRF). The dataset included 38 candidate feature variables, classified as follows:

Demographic and general information included age, gender, height, weight, body mass index (BMI), education level (junior high school or below, high school and above), household annual income (<80,000 RMB, ≥80,000 RMB), occupation (mental workers, physical workers), payment type (basic medical care, urban medical care), marital status (married, separated), body temperature, respiratory rate, heart rate, systolic blood pressure, and diastolic blood pressure.

Medical history: Hypertension, diabetes mellitus, hyperlipidemia, hyperhomocysteinemia, prior transient ischemic attack (TIA), ongoing smoking and alcohol consumption.

Hematological and biochemical parameters, including red blood cell count (RBC), white blood cell count (WBC), platelet count (PLT), hemoglobin concentration (HGB), total cholesterol (TC), triglycerides (TG), low-density lipoprotein (LDL), high-density lipoprotein (HDL), prothrombin time (PT), fibrinogen content (FIB), activated partial thromboplastin time (APTT), thrombin time (TT), glycated hemoglobin (HbA1c), creatinine (Cr), uric acid (UA), and homocysteine (Hcy), were assessed.

To ensure data accuracy, all variables underwent validation by cross-referencing with primary reports. Missing or outlier values were addressed following predefined quality control protocols, such as mean imputation for moderately missing data and exclusion for variables with high rates of missing values.

### Outcome definition

The main outcome assessed in this study was the occurrence of CHD, which was characterized by a history of myocardial infarction, coronary artery stenosis of ≥50% as verified by angiography, or a clinical diagnosis of angina pectoris substantiated by electrocardiographic and biomarker findings.

## Methods

### Data collection and preprocessing

All patients entered the cohort at the time of their index clinical evaluation for carotid atherosclerosis, defined as the first documented carotid ultrasound or CTA performed between January 1, 2022 and July 20, 2025 at the First Affiliated Hospital of Henan University of Chinese Medicine. Demographic information, laboratory measurements, vital signs, and medical history variables were all obtained at this same index encounter, ensuring that all predictors reflected true baseline status. Medical history conditions (e.g., hypertension, diabetes, hyperlipidemia, prior stroke) were extracted from structured electronic medical record entries documented before or on the index date. These diagnoses were obtained from structured fields within the hospital’s electronic medical record system, which are internally mapped to ICD-10 diagnostic terminology (e.g., I10 for hypertension, E11 for diabetes, E78.5 for dyslipidemia, I63 for ischemic stroke), ensuring standardized and reproducible extraction.

Because this study followed a cross-sectional diagnostic design, the target outcome – presence or absence of CHD – represented each patient’s clinical status at the index date. No longitudinal follow-up, temporal prediction, or post-index data were used.

A total of 38 clinical variables – including demographics, medical history, lifestyle factors, laboratory indicators, and imaging-confirmation results – were retrieved from electronic medical records. Ultrasound and CTA findings were used solely to confirm the diagnosis of carotid atherosclerosis and were not incorporated as predictor variables due to inconsistent availability of detailed imaging metrics. Continuous variables were standardized using *z*-score normalization, and missing values were handled according to predefined rules. To prevent information leakage, all preprocessing steps were fitted only on the training set and applied unchanged to the validation and test sets.

**Figure 1.**
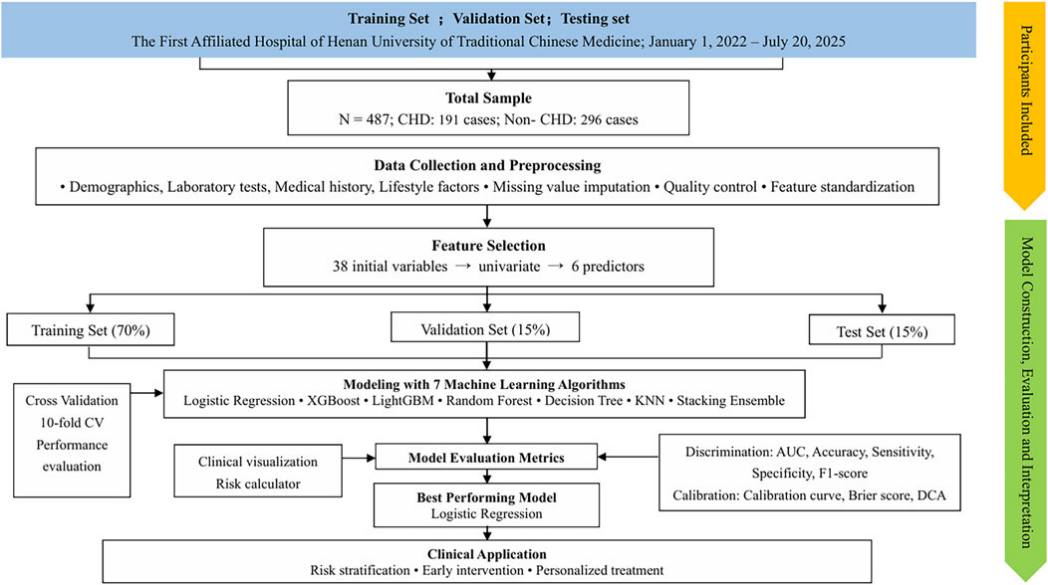
Flowchart of the study design and modeling pipeline. The pipeline included sequential steps: dataset partitioning (training/validation/test), training-set–based preprocessing, training-set–based feature selection, model training with cross-validation, validation-set threshold optimization, final evaluation on the independent test set, and SHAP/DCA analyses.

To ensure methodological rigor and reproducibility, the modeling workflow followed a strictly sequential pipeline (Figure 1). After preprocessing, the dataset was randomly split into training (70%), validation (15%), and test (15%) subsets through stratified sampling to preserve outcome prevalence. Feature selection was restricted to the training set, beginning with univariate screening followed by LASSO regression with 10-fold cross-validation. Hyperparameters for all ML models were tuned using five-fold cross-validation within the training set. The validation set was used exclusively for probability threshold optimization via the Youden index, and final model performance was evaluated on the independent test set.

### Dataset partitioning and class imbalance management

To enhance reproducibility and mitigate sampling bias, the dataset underwent stratified random splitting into training (70%), validation (15%), and test (15%) subsets, with a fixed random seed (random_state = 2024). To tackle the moderate class imbalance (CHD: non-CHD ≈ 1:1.55), compatible models such as Logistic regression model, Decision Tree, and Random Forest were trained using inverse class weighting (class_weight = “balanced”). In the case of gradient boosting models like XGBoost and LightGBM, scale_pos_weight was adjusted to approximately 1.55 to address the imbalance (296/191 ≈ 1.55).

### Model construction and hyperparameter optimization

Seven ML algorithms were developed: logistic regression, decision tree, Random Forest, K-nearest neighbors (KNN), XGBoost, LightGBM, and a stacking ensemble. Hyperparameters were optimized via five-fold cross-validation performed exclusively within the training set, with AUC as the primary selection metric (Supplementary Table 1).

The stacking ensemble incorporated the six individual models as base learners and logistic regression as the meta-learner. Out-of-fold predictions generated during cross-validation were used to train the meta-learner, ensuring that no information from the validation or test sets influenced its training.

All analyses were conducted in Python using scikit-learn, xgboost, and lightgbm libraries, with fixed random seeds to ensure reproducibility.

### Feature selection

The 38 initial candidate predictors were selected based on established coronary heart disease (CHD) risk factors reported in major cardiovascular guidelines (e.g., ESC 2023; ACC/AHA 2019) and prior literature, supplemented by expert clinical input from cardiologists and neurologists at our institution. A total of 38 candidate predictors were initially assessed. Univariate analyses were conducted within the training dataset to identify features with significant differences between CHD and non-CHD groups.

To reduce dimensionality and avoid overfitting, LASSO regression with 10-fold cross-validation was applied to the variables retained from univariate screening. This procedure identified six key predictors used for final model development. No feature selection steps involved the validation or test datasets to avoid information leakage.

### Model evaluation metrics

The model’s performance was thoroughly assessed across training, validation, and test datasets utilizing various metrics. Discrimination metrics included AUC, accuracy, sensitivity, specificity, and F1 score. Calibration was evaluated through Brier scores and calibration curves employing 10 equally populated bins. Precision-Recall (PR) analysis assessed model resilience to class imbalance by calculating average precision (AP). Clinical utility was determined through decision curve analysis (DCA) spanning threshold probabilities from 0.01 to 0.8.

Novel evaluation metrics were developed to calculate specificity and generate visual representations through the utilization of plotting tools such as matplotlib, seaborn, and scikit-learn.

Classification metrics that depend on a probability cutoff (e.g., accuracy, sensitivity, specificity, and F1 score) were calculated using thresholds optimized from the validation set rather than the default 0.5 threshold, in order to avoid information leakage from the test set.

### Model interpretability via SHAP

To improve transparency and facilitate clinical interpretation, the SHAP method was employed on the optimal Logistic Regression model. A LinearExplainer was instantiated with the training data, and SHAP values were computed on the test set. Various visual representations were produced, such as Summary plots (bar and beeswarm) and Waterfall plots to elucidate individualized prediction breakdowns.

These visual aids facilitated a comprehensive comprehension of both the global and local impacts of features on CHD risk.

### DCA

Decision curve analysis was performed on all models to assess the net clinical benefit at various probability thresholds. Threshold-specific net benefit values were computed, and reference lines for the “treat all” and “treat none” strategies were incorporated to provide a clinical decision-making context for the model’s utility. The outcomes of the decision curve analysis were documented in Excel format to ensure transparency and reproducibility.

### Software and reproducibility

The analyses were performed utilizing Python 3.13 on a Windows 11 platform. Key packages utilized encompassed pandas, numpy, scikit-learn, xgboost, lightgbm, matplotlib, and shap. Random processes were standardized through fixed seeds (random_state = 2024), and model training protocols were version-controlled to ensure reproducibility.

### Sample size and model deployment

The minimum sample size necessary for predictive modeling was determined following guidelines for developing clinical prediction models, considering the principles of events per variable (EPV), discrimination precision, and calibration requirements [15–17]. To ensure model stability and prevent overfitting in predicting CHD, a minimum of 10–20 outcome events per predictor variable was aimed for [18]. Following LASSO regularization, six variables were chosen, establishing an initial EPV range of 60–120 CHD cases.

To assess adequacy, we employed the pmsampsize *R* package (v1.1.4) to determine the minimum necessary sample size based on the following criteria: binary outcome; anticipated *R*^2^ = 0.15 (a conservative estimate for clinical data); 6 predictors; and an expected outcome prevalence of 30%. The analysis yielded a minimum required sample size of *N* = 368, with a minimum of 103 CHD events, to attain a shrinkage factor exceeding 0.9 and mitigate overfitting.

Furthermore, an AUC-based power analysis with a significance level of 0.05 and a power of 0.90, aiming to detect a clinically significant improvement in AUC from 0.50 to 0.60, indicated the need for a minimum of 95 cases with CHD and 240 CHD-negative cases, totaling 335 individuals [19]. The actual study population, consisting of 487 individuals with 191 CHD cases and 296 controls, exceeded these requirements, guaranteeing adequate power, model stability, and generalizability for both developing the model and comparing its performance.

## Results

### Univariate analysis and preliminary feature selection

A total of 487 patients diagnosed with carotid atherosclerosis participated in the study, with 191 individuals in the CHD cohort and 296 in the non-CHD control group. Initially, 38 candidate variables were gathered, covering demographic characteristics (e.g., age, sex, body mass index [BMI], education level), medical history (e.g., hypertension, diabetes mellitus, smoking status), biochemical markers (e.g., thrombin time (TT), triglycerides [TG], low-density lipoprotein [LDL], creatinine [Cr], and homocysteine [Hcy]), as well as specific vital signs (e.g., diastolic blood pressure, body temperature). Univariate analysis identified significant differences in variables such as age, BMI, diastolic blood pressure, thrombin time (TT), creatinine (Cr), and homocysteine (Hcy) between the CHD and non-CHD groups (refer to Table 1), indicating their potential relevance to CHD risk. These variables were subsequently incorporated into the feature selection and model development phase.

**Table 1.** Baseline characteristics of patients with and without CHD. Demographic, clinical, and biochemical parameters were compared between groups with and without CHD utilizing suitable statistical analyses. Continuous variables were expressed as mean ± standard deviation, while categorical variables were presented as frequencies (percentages). The reported *P* values signify the statistical significance observed between the two groups

Characteristic	Control group (*N* = 296)	Case group (*N* = 191)	*p*-value
Demographic and general information			
Age	63.36 ± 9.84	69.59 ± 8.44	0.00*
Gender			
Male	199 (0.64)	113 (0.36)	0.08
Female	97 (0.55)	78 (0.45)	
Height (cm)	167.42 ± 6.84	165.79 ± 7.56	0.02*
Weight (kg)	67.36 ± 9.84	67.76 ± 10.04	0.71
Body mass index (BMI)	23.98 ± 2.76	24.70 ± 3.06	0.01*
Education level			
Junior high school or below	220 (0.67)	109 (0.33)	0.00*
High school and above	71 (0.45)	82 (0.52)	
Household annual income (RMB 10,000 yuan)			
<8	259 (0.62)	157 (0.38)	0.15
8 ≤	37 (0.52)	34 (0.48)	
Occupation in the 6 months prior to onset			
Mental workers	32 (0.58)	23 (0.42)	0.48
Physical workers	264 (0.61)	168 (0.39)	
Payment type			
Basic medical care	113 (0.45)	137 (0.55)	0.00*
Urban medical care	183 (0.77)	54 (0.23)	
Marital status			
Married	281 (0.60)	185 (0.40)	0.31
Separated	15 (0.71)	6 (0.29)	
Body temperature	36.52 ± 0.29	36.44 ± 0.25	0.00*
Respiratory rate	18.85 ± 1.64	18.73 ± 1.91	0.05*
Heart rate	75.30 ± 11.24	77.12 ± 11.81	0.06
Systolic blood pressure	148.28 ± 21.02	142.95 ± 20.68	0.00*
Diastolic blood pressure	88.76 ± 12.71	80.88 ± 13.07	0.00*
Medical history			
Hypertension	154(0.51)	147(0.49)	0.00*
Diabetes mellitus	208(0.68)	97(0.32)	0.00*
Hyperlipidemia	29(0.45)	35 (0.55)	0.01*
Hyperhomocysteinemia	6(0.14)	38(0.86)	0.00*
History of TIA	24(0.57)	18(0.43)	0.61
Continuous smoking history	100(0.68)	46(0.32)	0.02*
Continuous drinking history	41(0.59)	29(0.41)	0.68
Blood biochemical indicators			
RBC - red blood cell count	4.48 ± 0.47	4.41 ± 0.54	0.10
WBC - white blood cell count	6.74 ± 2.19	6.28 ± 2.05	0.01*
PLT - platelet count	213.51 ± 58.24	203.35 ± 53	0.07
HGB - hemoglobin concentration	140.90 ± 28.36	138.38 ± 27.46	0.10
TC - total cholesterol	4.61 ± 1.13	4.18 ± 1.10	0.00*
TG - triglycerides	1.74 ± 1.13	1.46 ± 0.91	0.00*
LDL - low - density lipoprotein	2.78 ± 0.89	2.46 ± 0.88	0.00*
HDL - high - density lipoprotein	1.20 ± 0.31	1.20 ± 0.29	0.71
PT - prothrombin time	11.33 ± 3.38	11.36 ± 2.29	0.29
FIB - fibrinogen content	3.07 ± 0.84	3.20 ± 0.83	0.11
APTT - activated partial thromboplastin time	29.41 ± 5.02	29.83 ± 4.28	0.24
TT - thrombin time	19.73 ± 69.57	15.17 ± 1.72	0.00*
HbA1c - glycated hemoglobin	6.73 ± 1.91	6.71 ± 1.54	0.34
Cr - creatinine	66.12 ± 29.76	62.35 ± 20.56	0.05*
UA - uric acid	289.16 ± 84.32	297.91 ± 89.75	0.31
Hcy - homocysteine	15.71 ± 10.59	16.08 ± 7.64	0.01*

### Feature selection via LASSO regression

**Figure 2.**
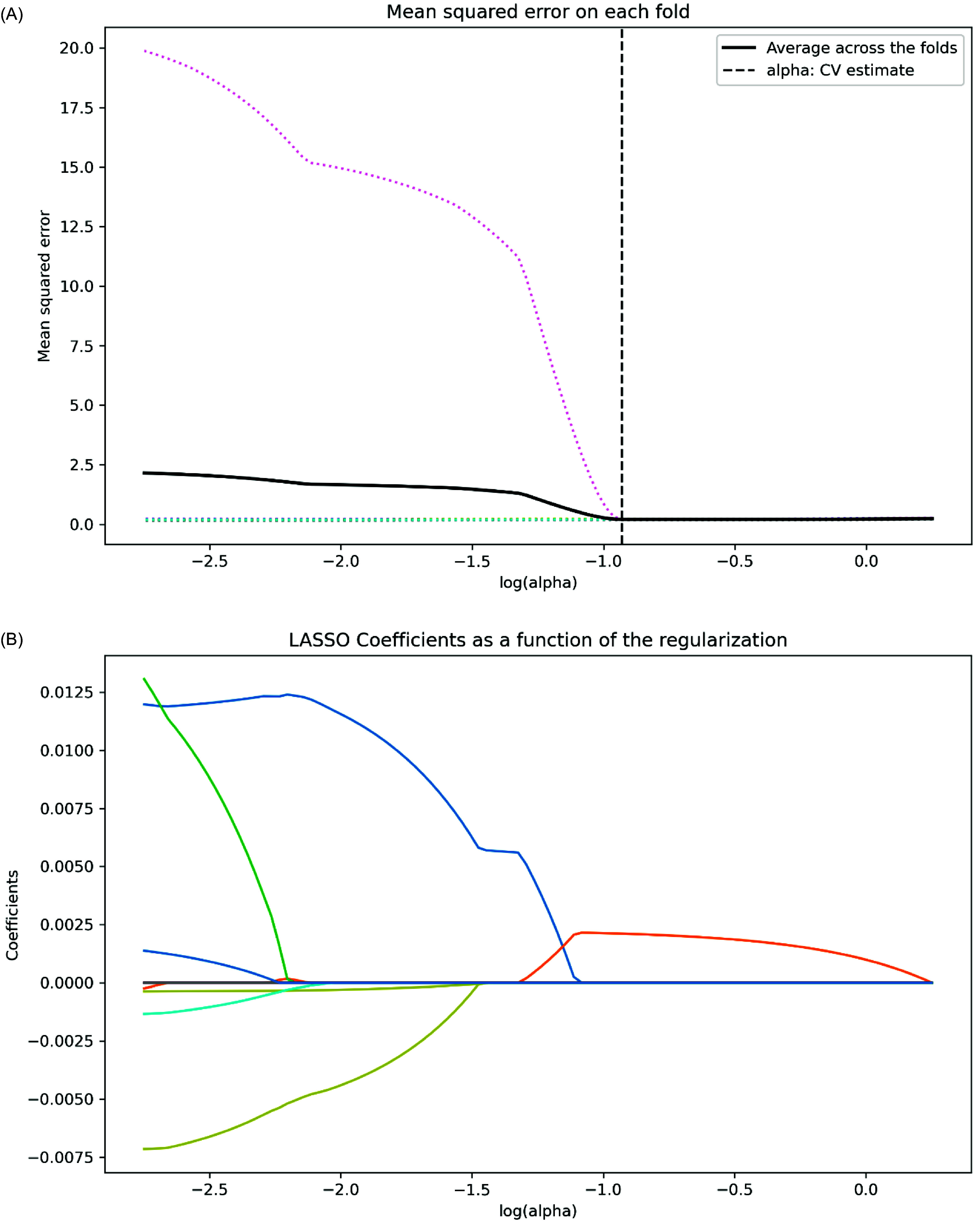
LASSO regression for feature selection. (A) Ten-fold cross-validation plot for selecting the optimal*λ*value minimizing mean squared error. (B) Coefficient trajectories of candidate features across different*λ*values, emphasizing the six ultimate predictors selected.

To enhance feature selection, decrease dimensionality, and alleviate overfitting risks, we employed LASSO regression on the initial set of 38 variables. The regularization parameter (*α* = 0.1174) was optimized through ten-fold cross-validation, aligning with the lowest mean squared error as illustrated in Figure 2A.

Six key predictors closely linked to CHD risk in individuals with carotid atherosclerosis were identified: age, BMI, diastolic blood pressure, thrombin time (TT), creatinine (Cr), and homocysteine (Hcy) (Figure 2B). These factors were utilized as input variables for developing a ML model, establishing a reliable basis for precise CHD risk assessment.

### Performance evaluation of machine learning models across training, validation, and testing sets discrimination performance

#### ROC curve analysis

**Figure 3.**
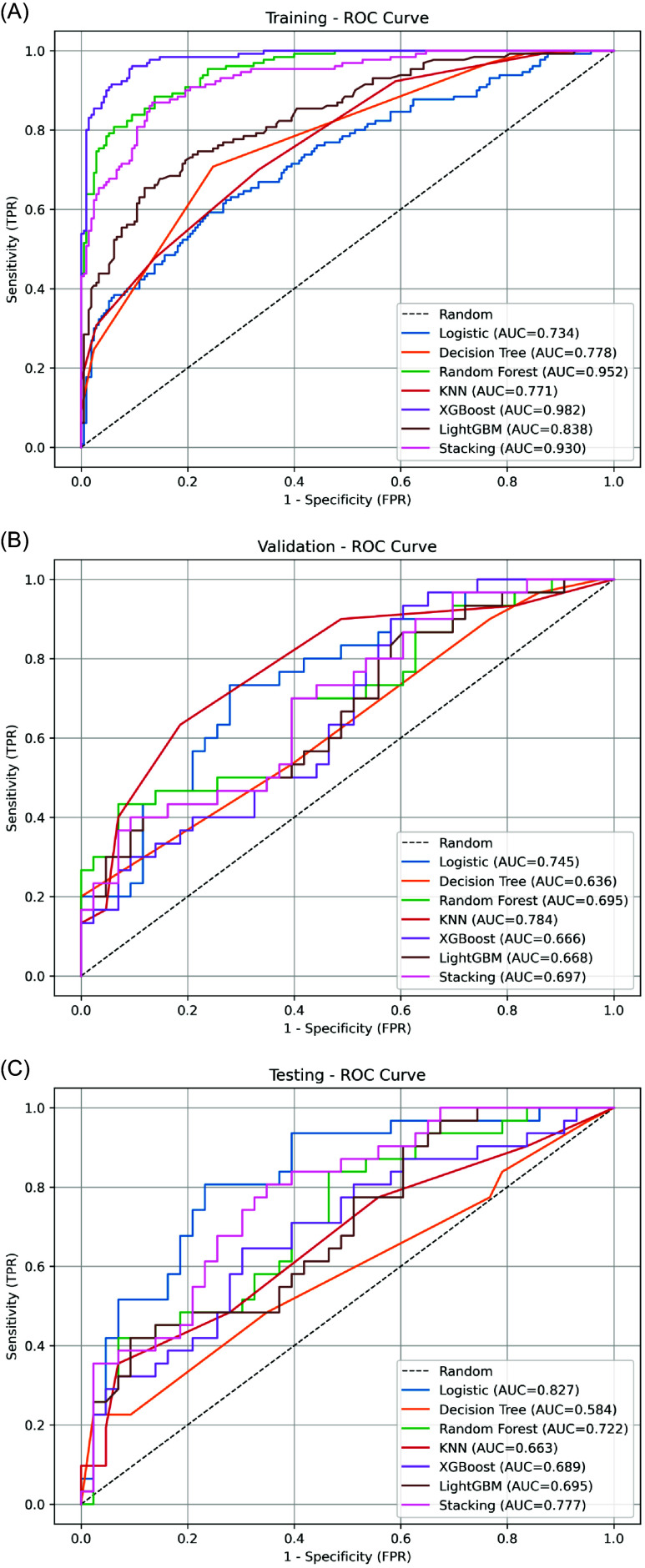
ROC curves of seven machine learning models across datasets. (A) Training set; (B) Validation set; (C) Testing set. Logistic regression model and ensemble models (e.g., XGBoost, Random Forest) exhibited strong discrimination in training but variable generalization performance in testing.

In the training dataset, ensemble models exhibited superior discriminative performance, with XGBoost and Random Forest showing the highest AUC values. Logistic regression model and KNN yielded comparatively lower AUCs (Figure 3A).

In the validation set, AUC values declined across all models, indicating varying degrees of overfitting. KNN and logistic regression model maintained relatively better discrimination, whereas Decision Tree showed the weakest performance (Figure 3B).

In the testing dataset, the logistic regression model demonstrated the best discrimination (AUC = 0.827), indicating strong generalizability. Stacking and Random Forest also achieved acceptable performance, while Decision Tree showed limited discriminatory capability (Figure 3C; Supplementary Table 2).

### Classification performance

#### Precision-recall curve (PRC) analysis

**Figure 4.**
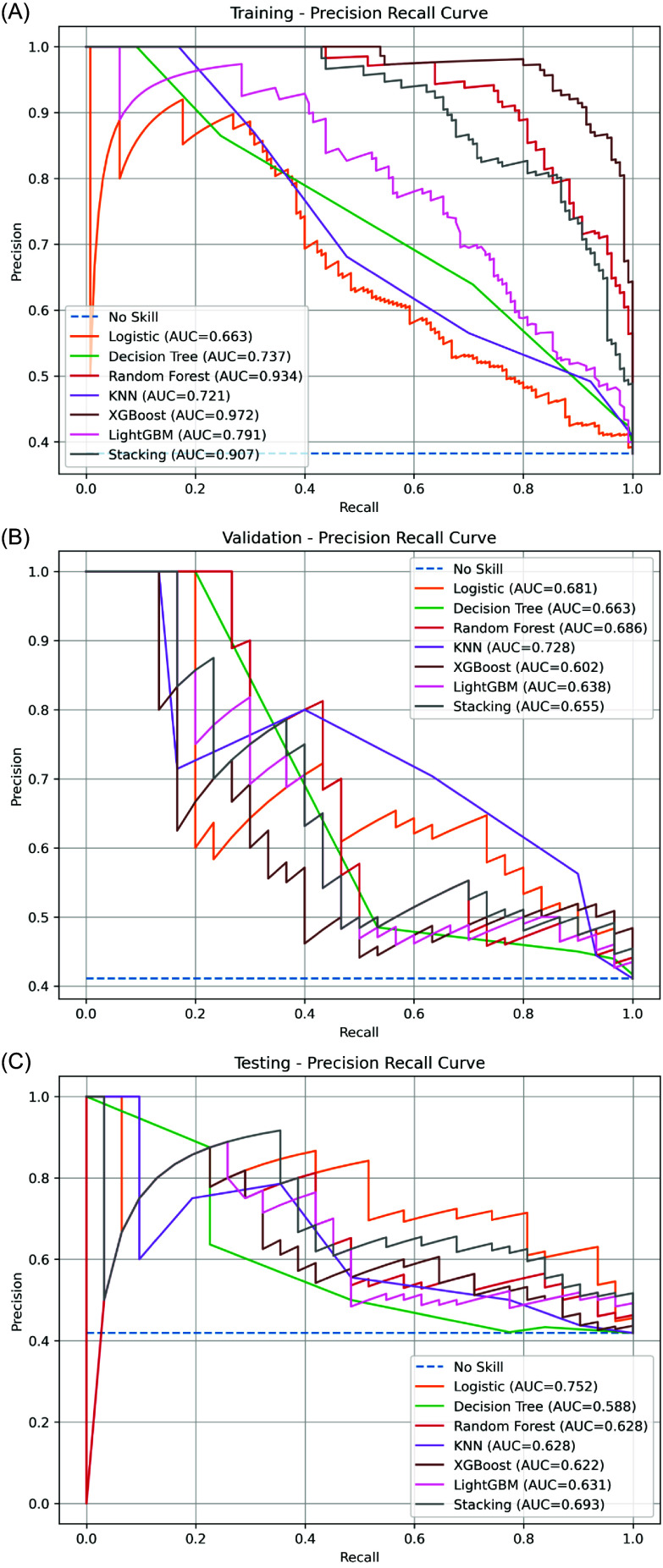
PR curves of seven machine learning models. (A) Training set. (B) Validation set. (C) Testing set. Logistic regression model achieved the highest PRC-AUC in the testing set, indicating balanced precision and recall under real-world conditions.

Patterns in the PRC analysis are generally consistent with the ROC findings. In the training set, ensemble models again demonstrated strong performance, while logistic regression model ranked lower (Figure 4A).

In the validation set, all models showed reduced area under the precision–recall curve (PRC-AUC) values. KNN, Random Forest, and logistic regression model provided comparatively better balance between precision and recall, whereas XGBoost exhibited a notable performance drop (Figure 4B).

In the testing dataset, the logistic regression model achieved the best overall PRC-AUC performance, whereas Random Forest and XGBoost demonstrated reduced precision under real-world conditions (Figure 4C).

### Calibration performance

**Figure 5.**
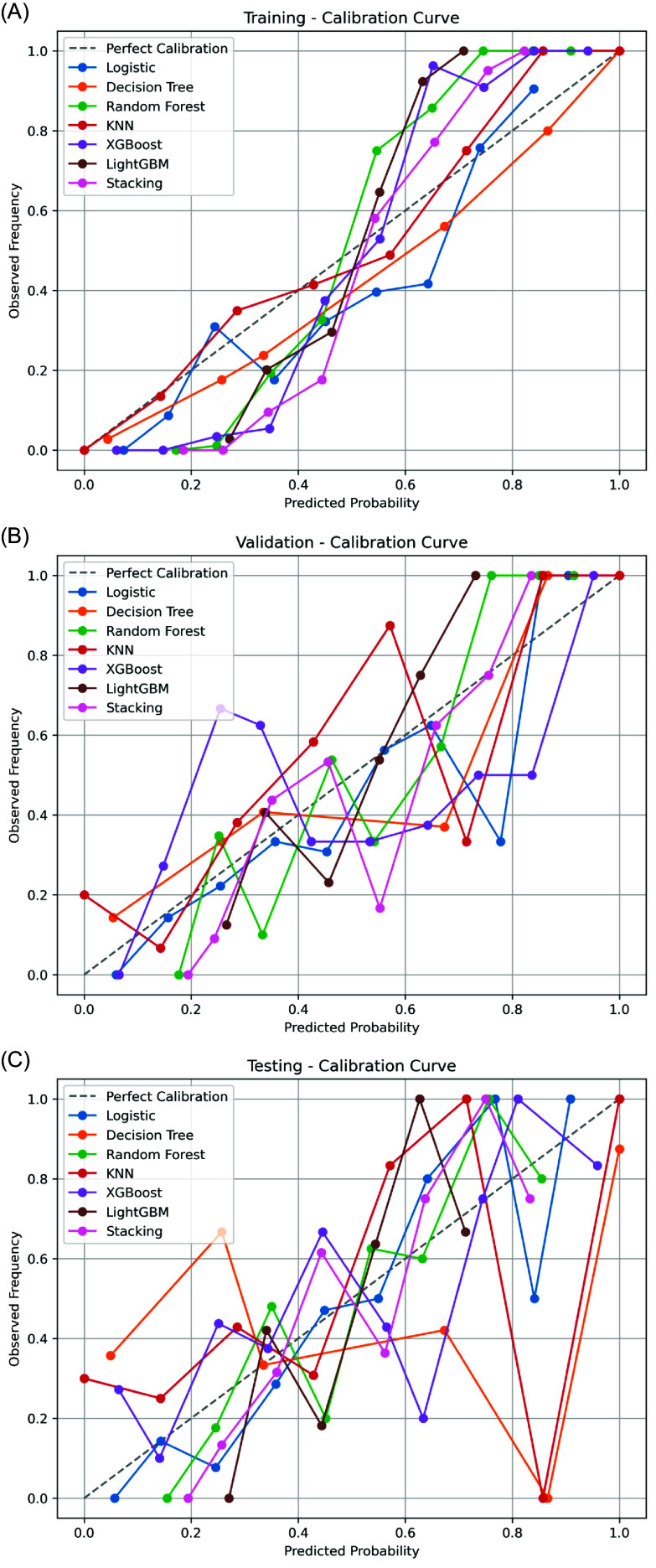
Calibration curves of machine learning models. (A) Training set. (B) Validation set. (C) Testing set. Logistic regression model, Random Forest, and XGBoost showed relatively good alignment between predicted and observed probabilities in the testing set.

Across the training dataset, LightGBM, XGBoost, and Stacking exhibited strong calibration, closely approximating the ideal diagonal line (Figure 5A).

In the validation set, calibration curves varied considerably, suggesting greater uncertainty in predicted probabilities. Only logistic regression model and LightGBM showed moderate concordance with observed outcomes (Figure 5B).

In the testing dataset, logistic regression model, Random Forest, and XGBoost demonstrated more stable calibration, while Decision Tree and KNN exhibited overestimation or underestimation across different probability ranges (Figure 5C).

### Model interpretability and clinical utility

**Figure 6.**
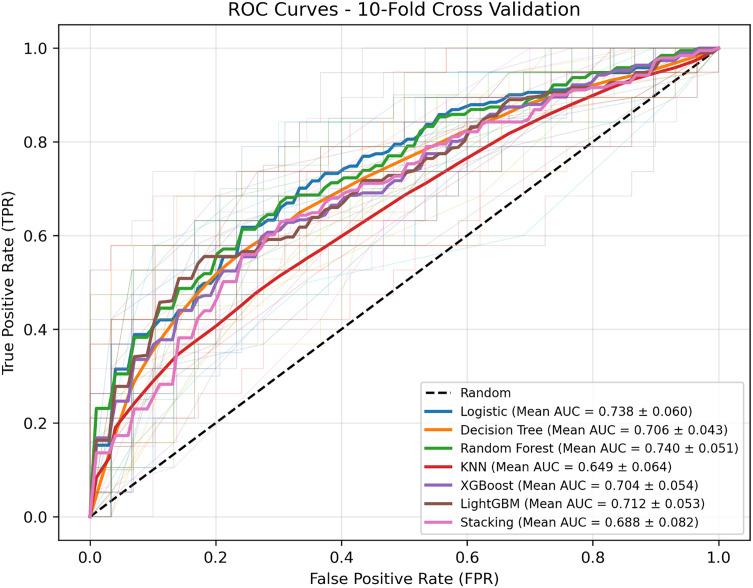
Ten-fold cross-validation results for all models. Bar plots display the mean AUC with standard deviation for each model across ten validation folds. Logistic regression model and Random Forest achieved the highest average AUCs (0.738 and 0.740, respectively).

To assess overall model stability, a 10-fold cross-validation was conducted during training. All models achieved mean AUC values above 0.65, with random forest and logistic regression model showing the most consistent performance, while Decision Tree, KNN, and Stacking displayed greater variability (Figure 6).

**Figure 7.**
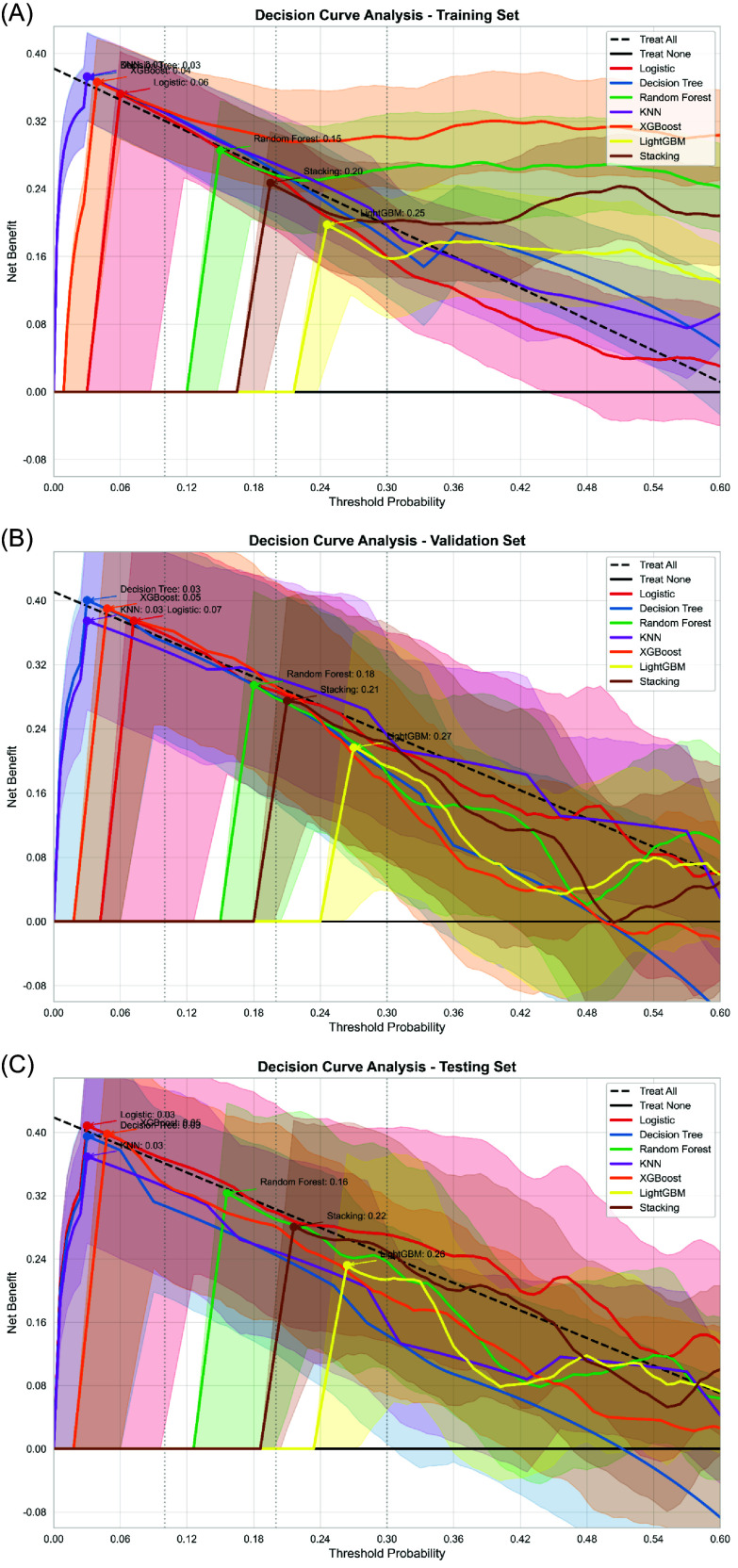
DCA of the logistic regression model. (A) Training set. (B) Validation set. (C) Testing set. The net benefit across different probability thresholds is illustrated. Logistic regression model consistently outperformed or closely matched other models, confirming its clinical utility.

Regarding clinical applicability, DCA demonstrated that the logistic regression model consistently yielded favorable net benefit across the training, validation, and testing cohorts (Figure 7). Although LightGBM achieved slightly higher benefit at certain thresholds, its performance was less stable, reinforcing the practical advantages of logistic regression model.

Based on its balanced discrimination, calibration, and interpretability, the logistic regression model was selected as the final recommended model. SHAP was then applied to enhance model transparency and evaluate feature contributions at both the individual and global levels.

**Figure 8.**
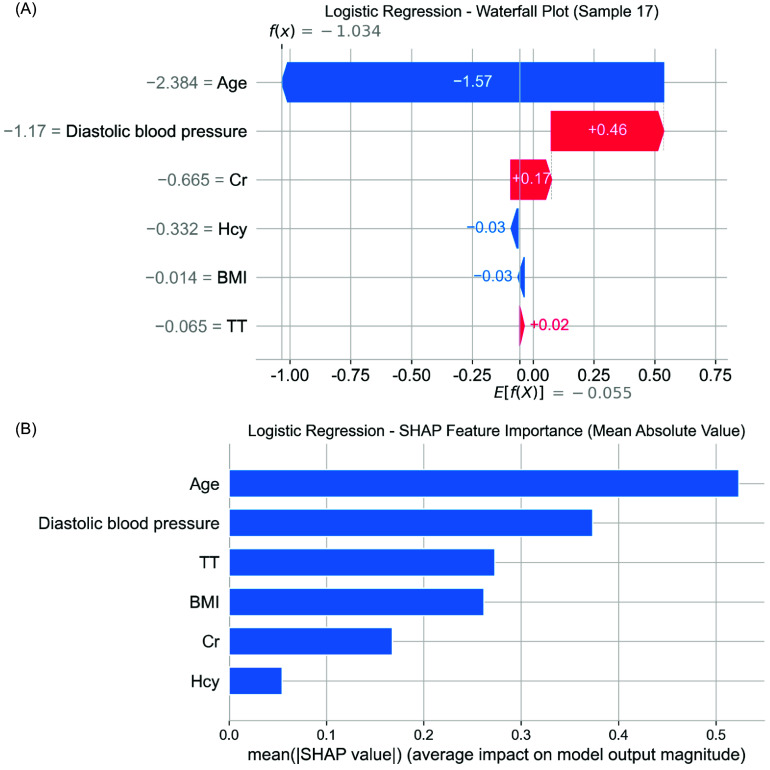
(A) SHAP waterfall plot for individual prediction (Sample 17). The figure shows the contribution of each feature to the CHD prediction for a representative patient. Age and diastolic blood pressure exerted the strongest negative influence, while creatinine and thrombin time had positive contributions. (B) SHAP summary bar plot of feature importance. Mean absolute SHAP values across all testing samples are presented, ranking features by their overall impact on model output. Age, diastolic blood pressure, and thrombin time were the most influential predictors.

At the individual level, the SHAP waterfall plot revealed that age and diastolic blood pressure exerted the largest negative contributions to predicted CHD risk, while creatinine, thrombin time, and homocysteine increased risk probability (Figure 8A).

At the global level, SHAP summary plots confirmed age and diastolic blood pressure as the most influential predictors, followed by thrombin time (TT), BMI, creatinine (Cr), and homocysteine (Hcy). The ranking of SHAP importance aligned with logistic regression model coefficients, supporting the biological plausibility and interpretability of the model (Figure 8B).

Overall, the logistic regression model demonstrated strong predictive performance, stable generalization, and high interpretability, supporting its potential use in early identification and personalized risk assessment of CHD among patients with carotid atherosclerosis.

### Supplementary threshold optimization analysis

To further explore clinically meaningful probability cutoffs, we conducted a supplementary threshold optimization analysis using the validation dataset. For each model, thresholds were varied across a clinically relevant range, and the optimal cutoff was defined according to the Youden index (sensitivity + specificity − 1). This procedure ensured that threshold selection was performed without accessing the test data, thereby preventing information leakage.

As an illustrative example, the optimal threshold for the random forest model shifted from the default 0.50 to 0.55 based on validation-set performance, which improved precision and the F1 score at the expense of sensitivity. These findings indicate that different models may be better suited for distinct clinical priorities (e.g., minimizing false positives vs. maximizing sensitivity). All classification metrics reported in the main text therefore reflect thresholds derived from the validation set rather than any fixed default value(see Supplementary Table 3).

## Discussion

This study developed and compared multiple ML models to predict CHD in individuals with carotid atherosclerosis, with a focus on model interpretability and clinical applicability. Overall, a parsimonious logistic regression model demonstrated comparable or superior performance to more complex ensemble algorithms and was therefore selected as the final predictive model. Using LASSO regression, six clinically relevant predictors were identified, and subsequent SHAP analysis confirmed their biological plausibility and relative contribution to risk stratification. Furthermore, decision curve analysis indicated that the logistic regression model provided a consistent net clinical benefit across a broad range of threshold probabilities, supporting its potential utility as a practical tool for CHD risk assessment in this population.

## Key findings in context

Logistic regression model outperformed complex ensemble models such as XGBoost, which exhibited substantial overfitting despite high training performance. This observation is consistent with the principle of Occam’s razor, which suggests that simpler models often generalize better in datasets of moderate size with low-to-moderate feature dimensionality [20]. The overfitting seen in XGBoost and Random Forest models may be attributed to their capacity to memorize noise present in the training data, underscoring the importance of prioritizing real-world utility over training accuracy [21].

The six features selected by the LASSO method exhibited clear biological plausibility [22]. Age, a well-established risk factor for CHD, was identified by SHAP analysis as the most influential variable. However, its negative SHAP value in specific instances (e.g., Sample 17) suggests context-specific effects, possibly indicating nonlinear relationships in elderly patients with carotid atherosclerosis. The inverse relationship between diastolic blood pressure and CHD risk in this particular cohort contradicts findings from general population studies [8] but may be attributed to the distinct hemodynamic profile of individuals with carotid stenosis, where lower diastolic pressure could potentially mitigate carotid plaque burden [5].

Biochemical markers provide additional evidence supporting pathogenic connections: increased levels of creatinine indicate compromised renal function, a recognized risk factor for CHD through endothelial dysfunction [23]; elevated homocysteine levels contribute to oxidative stress and vascular inflammation [24]; and prolonged thrombin time may indicate coagulation abnormalities [25], which are implicated in atherothrombosis. These correlations strengthen the credibility of the model and offer mechanistic explanations for the co-occurrence of CHD in carotid atherosclerosis.

## Comparison with existing literature

Our study contributes to the existing literature in three key aspects. Firstly, although previous research has demonstrated the superior performance of ensemble models over traditional approaches in predicting CHD [20], our results underscore the continued relevance of simpler models in specific subpopulations (e.g., individuals with carotid atherosclerosis) owing to their reliability and ease of interpretation. This finding aligns with recent observations by Evangelou et al [26], who highlighted the variability in ML model efficacy across different populations and scenarios.

The novel identification of TT as a significant predictor distinguishes this study. While many CHD risk models typically emphasize prothrombin time (PT) or INR [27], our findings underscore the clinical relevance of thrombin time, a marker of fibrinogen functionality. This observation may underscore the potential impact of fibrinogen on the advancement of carotid plaque and the development of CHD, necessitating additional research [28].

Thirdly, SHAP-based interpretability fills a crucial void in ML research concerning cardiovascular diseases. In contrast to research that merely presents model performance metrics without providing detailed explanations at the feature level [29], our investigation precisely determines the impact of each variable on predictions. For instance, we elucidate the dual nature of age as a risk factor in general but as a protective factor in certain instances. This level of transparency is imperative for gaining acceptance among clinicians and facilitating the clinical application of our findings [30].

## Clinical implications

The robust performance of the logistic regression model, combined with its consistent net benefit in DCA, supports its clinical utility for stratifying CHD risk in patients with carotid atherosclerosis. Importantly, the model relies on six routinely collected clinical variables – age, BMI, blood pressure, total cholesterol, creatinine, and homocysteine – making it practical for real-world integration without requiring advanced imaging or specialized diagnostic resources. This simplicity enhances its potential for use in primary care and resource-limited settings, enabling timely and accessible risk assessment.

The model’s interpretability facilitates personalized risk communication. For instance, a patient exhibiting elevated levels of creatinine (Cr) and homocysteine (Hcy) could receive guidance on managing renal function and supplementing with B-vitamins to lower Hcy levels [31]. Similarly, individuals with a high body mass index (BMI) could benefit from tailored advice on weight management. These practical insights serve to connect predictive modeling with preventive healthcare.

## Limitations

This study has several limitations. First, its retrospective single-center design may introduce selection bias and limit generalizability to broader populations. Second, mean imputation was applied to handle missing data, which may underestimate variability; more robust methods such as multiple imputation should be considered in future research [32].

Third, although major cardiovascular guidelines recommend additional predictors – such as family history of premature CHD, physical activity, C-reactive protein (CRP), lipoprotein(a), and apolipoprotein B – their inclusion was limited by real-world data constraints. Many of these variables were either unavailable, inconsistently recorded, or exhibited high missingness within the electronic medical record system, and were therefore excluded to avoid introducing bias. Importantly, all available candidate predictors were subjected to standardized feature selection procedures, and only variables identified as stable contributors through univariate analysis and LASSO regression were retained.

Fourth, several potentially relevant factors – such as medication history, carotid plaque morphology, and treatment adherence – were not available, which may have affected predictive performance. Finally, the model has yet to be tested in external cohorts; prospective multicenter validation will be essential before clinical implementation [17].

## Future directions

Future research should focus on several key areas: (1) validating the model across multiple centers using prospective cohorts to ensure broader generalizability; (2) incorporating additional data modalities – such as carotid ultrasound–derived imaging markers and relevant genetic indicators –to further enhance predictive precision as data availability improves; (3) exploring dynamic threshold optimization tailored to different clinical scenarios (e.g., screening versus secondary prevention); and (4) developing user-friendly tools, such as web-based or electronic health record–integrated calculators, to facilitate seamless clinical implementation.

In conclusion, this study shows that a concise logistic regression model, constructed using biologically relevant predictors, provides reliable CHD risk assessment in patients with carotid atherosclerosis. The model’s interpretability and clinical usefulness render it a valuable instrument for individualized risk assessment, underscoring the significance of harmonizing model intricacy with practical relevance in cardiovascular investigations.

## Supporting information

10.1017/cts.2026.10722.sm001 Zhang et al. supplementary material 1 Zhang et al. supplementary material

10.1017/cts.2026.10722.sm002 Zhang et al. supplementary material 2 Zhang et al. supplementary material

10.1017/cts.2026.10722.sm003 Zhang et al. supplementary material 3 Zhang et al. supplementary material

10.1017/cts.2026.10722.sm004 Zhang et al. supplementary material 4 Zhang et al. supplementary material

## Data Availability

The datasets produced and/or analyzed in this study are not publicly accessible due to patient confidentiality and institutional guidelines; however, they can be obtained from the corresponding author upon request.

## Supplementary material

The supplementary material for this article can be found at https://doi.org/10.1017/cts.2026.10722.
