# Supplementary material for: Explainable machine learning for predicting coronary heart disease risk in patients with carotid atherosclerosis: A retrospective study with SHAP and decision curve analysis
Source: J Clin Transl Sci. 2026 Mar 6;10(1):e57. doi: 10.1017/cts.2026.10722 (PMC13058763; doi:10.1017/cts.2026.10722)
Supplement: Zhang et al. supplementary material 1 — Zhang et al. supplementary material [file S2059866126107225sup001.docx]

**Supplementary Table 1.** Optimized hyperparameters for the seven machine learning models.

| **Model** | **Hyperparameters Used After GridSearchCV** |
| --- | --- |
| Logistic Regression | Penalty = L2; C = 0.1; class_weight = ‘balanced’ |
| Decision Tree | max_depth = 5; min_samples_split = 5 |
| Random Forest | n_estimators = 100; max_depth = 7 |
| K-Nearest Neighbors (KNN) | n_neighbors = 5; weights = ‘uniform’ |
| XGBoost | max_depth = 5; learning_rate = 0.1; subsample = 1.0; eval_metric = ‘logloss’ |
| LightGBM | max_depth = 5; learning_rate = 0.1; boosting_type = ‘gbdt’ |
| Stacking Ensemble | Base learners: six individual models; Meta-learner: logistic regression (trained on out-of-fold predictions) |

This table summarizes the optimal hyperparameters selected for each machine learning model based on five-fold cross-validation performed exclusively within the training set. GridSearchCV was used to identify the parameter combinations that maximized the area under the ROC curve (AUC). The listed hyperparameters represent the final settings used for model training before validation-set threshold optimization and test-set performance evaluation.
