# Supplementary material for: Explainable machine learning for predicting coronary heart disease risk in patients with carotid atherosclerosis: A retrospective study with SHAP and decision curve analysis
Source: J Clin Transl Sci. 2026 Mar 6;10(1):e57. doi: 10.1017/cts.2026.10722 (PMC13058763; doi:10.1017/cts.2026.10722)
Supplement: Zhang et al. supplementary material 2 — Zhang et al. supplementary material [file S2059866126107225sup002.docx]

**Supplementary Table 2.** Performance metrics of seven machine learning models on the testing set.

| **Model** | **Stage** | **AUC** | **Accuracy** | **Sensitivity** | **Specificity** | **F1 Score** | **Brier Score** |
| --- | --- | --- | --- | --- | --- | --- | --- |
| Logistic | Training | 0.73 | 0.66 | 0.67 | 0.65 | 0.60 | 0.21 |
| Logistic | Validation | 0.74 | 0.70 | 0.67 | 0.72 | 0.65 | 0.20 |
| Logistic | Testing | 0.83 | 0.73 | 0.61 | 0.81 | 0.66 | 0.17 |
| Decision Tree | Training | 0.78 | 0.74 | 0.71 | 0.75 | 0.67 | 0.18 |
| Decision Tree | Validation | 0.64 | 0.58 | 0.53 | 0.60 | 0.51 | 0.24 |
| Decision Tree | Testing | 0.58 | 0.58 | 0.48 | 0.65 | 0.49 | 0.28 |
| Random Forest | Training | 0.95 | 0.89 | 0.78 | 0.95 | 0.84 | 0.12 |
| Random Forest | Validation | 0.69 | 0.63 | 0.47 | 0.74 | 0.51 | 0.21 |
| Random Forest | Testing | 0.72 | 0.69 | 0.45 | 0.86 | 0.55 | 0.21 |
| KNN | Training | 0.77 | 0.71 | 0.48 | 0.86 | 0.56 | 0.18 |
| KNN | Validation | 0.78 | 0.71 | 0.40 | 0.93 | 0.53 | 0.19 |
| KNN | Testing | 0.66 | 0.69 | 0.35 | 0.93 | 0.49 | 0.24 |
| XGBoost | Training | 0.98 | 0.93 | 0.90 | 0.95 | 0.91 | 0.08 |
| XGBoost | Validation | 0.67 | 0.58 | 0.47 | 0.65 | 0.47 | 0.24 |
| XGBoost | Testing | 0.69 | 0.62 | 0.42 | 0.77 | 0.48 | 0.23 |
| LightGBM | Training | 0.84 | 0.79 | 0.65 | 0.88 | 0.71 | 0.18 |
| LightGBM | Validation | 0.67 | 0.67 | 0.43 | 0.84 | 0.52 | 0.22 |
| LightGBM | Testing | 0.70 | 0.69 | 0.42 | 0.88 | 0.53 | 0.22 |
| Logistic | Training | 0.73 | 0.66 | 0.67 | 0.65 | 0.60 | 0.21 |
| Logistic | Validation | 0.74 | 0.70 | 0.67 | 0.72 | 0.65 | 0.20 |
| Logistic | Testing | 0.83 | 0.73 | 0.61 | 0.81 | 0.66 | 0.17 |
| Decision Tree | Training | 0.78 | 0.74 | 0.71 | 0.75 | 0.67 | 0.18 |
| Decision Tree | Validation | 0.64 | 0.58 | 0.53 | 0.60 | 0.51 | 0.24 |
| Decision Tree | Testing | 0.58 | 0.58 | 0.48 | 0.65 | 0.49 | 0.28 |

Metrics such as AUC and PRC-AUC are threshold-independent and are reported directly. For classification-based metrics (accuracy, sensitivity, specificity, F1 score, and precision), thresholds were derived from the optimal cutoff identified in the validation set using the Youden index. No test-set information was used during threshold determination.
