# Supplementary material for: Explainable machine learning for predicting coronary heart disease risk in patients with carotid atherosclerosis: A retrospective study with SHAP and decision curve analysis
Source: J Clin Transl Sci. 2026 Mar 6;10(1):e57. doi: 10.1017/cts.2026.10722 (PMC13058763; doi:10.1017/cts.2026.10722)
Supplement: Zhang et al. supplementary material 3 — Zhang et al. supplementary material [file S2059866126107225sup003.docx]

**Supplementary Table 3.** Threshold optimization results across multiple classification thresholds based on validation-set performance.

| **Threshold** | **Accuracy** | **Sensitivity** | **Specificity** | **F1 Score** | **Precision** | **Model** |
| --- | --- | --- | --- | --- | --- | --- |
| 0.1 | 0.405 | 1 | 0.022 | 0.569 | 0.397 | Logistic |
| 0.15 | 0.432 | 1 | 0.067 | 0.58 | 0.408 | Logistic |
| 0.2 | 0.473 | 1 | 0.133 | 0.598 | 0.426 | Logistic |
| 0.25 | 0.541 | 0.966 | 0.267 | 0.622 | 0.459 | Logistic |
| 0.3 | 0.568 | 0.931 | 0.333 | 0.628 | 0.474 | Logistic |
| 0.35 | 0.581 | 0.862 | 0.4 | 0.617 | 0.481 | Logistic |
| 0.4 | 0.595 | 0.828 | 0.444 | 0.615 | 0.49 | Logistic |
| 0.45 | 0.554 | 0.69 | 0.467 | 0.548 | 0.455 | Logistic |
| 0.5 | 0.635 | 0.621 | 0.644 | 0.571 | 0.529 | Logistic |
| 0.55 | 0.73 | 0.552 | 0.844 | 0.615 | 0.696 | Logistic |
| 0.6 | 0.716 | 0.379 | 0.933 | 0.512 | 0.786 | Logistic |
| 0.65 | 0.689 | 0.276 | 0.956 | 0.41 | 0.8 | Logistic |
| 0.7 | 0.689 | 0.276 | 0.956 | 0.41 | 0.8 | Logistic |
| 0.75 | 0.662 | 0.138 | 1 | 0.242 | 1 | Logistic |
| 0.8 | 0.662 | 0.138 | 1 | 0.242 | 1 | Logistic |
| 0.85 | 0.635 | 0.069 | 1 | 0.129 | 1 | Logistic |
| 0.1 | 0.392 | 1 | 0 | 0.563 | 0.392 | Decision Tree |
| 0.15 | 0.392 | 1 | 0 | 0.563 | 0.392 | Decision Tree |
| 0.2 | 0.527 | 0.828 | 0.333 | 0.578 | 0.444 | Decision Tree |
| 0.25 | 0.527 | 0.828 | 0.333 | 0.578 | 0.444 | Decision Tree |
| 0.3 | 0.527 | 0.828 | 0.333 | 0.578 | 0.444 | Decision Tree |
| 0.35 | 0.595 | 0.724 | 0.511 | 0.583 | 0.488 | Decision Tree |
| 0.4 | 0.595 | 0.724 | 0.511 | 0.583 | 0.488 | Decision Tree |
| 0.45 | 0.595 | 0.586 | 0.6 | 0.531 | 0.486 | Decision Tree |
| 0.5 | 0.595 | 0.586 | 0.6 | 0.531 | 0.486 | Decision Tree |
| 0.55 | 0.595 | 0.586 | 0.6 | 0.531 | 0.486 | Decision Tree |
| 0.6 | 0.595 | 0.586 | 0.6 | 0.531 | 0.486 | Decision Tree |
| 0.65 | 0.703 | 0.31 | 0.956 | 0.45 | 0.818 | Decision Tree |
| 0.7 | 0.703 | 0.31 | 0.956 | 0.45 | 0.818 | Decision Tree |
| 0.75 | 0.703 | 0.31 | 0.956 | 0.45 | 0.818 | Decision Tree |
| 0.8 | 0.703 | 0.31 | 0.956 | 0.45 | 0.818 | Decision Tree |
| 0.85 | 0.703 | 0.31 | 0.956 | 0.45 | 0.818 | Decision Tree |
| 0.1 | 0.405 | 1 | 0.022 | 0.569 | 0.397 | Random Forest |
| 0.15 | 0.432 | 1 | 0.067 | 0.58 | 0.408 | Random Forest |
| 0.2 | 0.432 | 0.931 | 0.111 | 0.562 | 0.403 | Random Forest |
| 0.25 | 0.486 | 0.828 | 0.267 | 0.558 | 0.421 | Random Forest |
| 0.3 | 0.595 | 0.793 | 0.467 | 0.605 | 0.489 | Random Forest |
| 0.35 | 0.595 | 0.724 | 0.511 | 0.583 | 0.488 | Random Forest |
| 0.4 | 0.662 | 0.621 | 0.689 | 0.59 | 0.562 | Random Forest |
| 0.45 | 0.689 | 0.517 | 0.8 | 0.566 | 0.625 | Random Forest |
| 0.5 | 0.716 | 0.448 | 0.889 | 0.553 | 0.722 | Random Forest |
| 0.55 | 0.743 | 0.448 | 0.933 | 0.578 | 0.812 | Random Forest |
| 0.6 | 0.743 | 0.379 | 0.978 | 0.537 | 0.917 | Random Forest |
| 0.65 | 0.689 | 0.241 | 0.978 | 0.378 | 0.875 | Random Forest |
| 0.7 | 0.676 | 0.207 | 0.978 | 0.333 | 0.857 | Random Forest |
| 0.75 | 0.676 | 0.207 | 0.978 | 0.333 | 0.857 | Random Forest |
| 0.8 | 0.676 | 0.207 | 0.978 | 0.333 | 0.857 | Random Forest |
| 0.85 | 0.662 | 0.172 | 0.978 | 0.286 | 0.833 | Random Forest |
| 0.1 | 0.5 | 0.931 | 0.222 | 0.593 | 0.435 | KNN |
| 0.15 | 0.5 | 0.931 | 0.222 | 0.593 | 0.435 | KNN |
| 0.2 | 0.581 | 0.931 | 0.356 | 0.635 | 0.482 | KNN |
| 0.25 | 0.581 | 0.931 | 0.356 | 0.635 | 0.482 | KNN |
| 0.3 | 0.635 | 0.69 | 0.6 | 0.597 | 0.526 | KNN |
| 0.35 | 0.635 | 0.69 | 0.6 | 0.597 | 0.526 | KNN |
| 0.4 | 0.595 | 0.483 | 0.667 | 0.483 | 0.483 | KNN |
| 0.45 | 0.595 | 0.483 | 0.667 | 0.483 | 0.483 | KNN |
| 0.5 | 0.662 | 0.345 | 0.867 | 0.444 | 0.625 | KNN |
| 0.55 | 0.689 | 0.31 | 0.933 | 0.439 | 0.75 | KNN |
| 0.6 | 0.689 | 0.31 | 0.933 | 0.439 | 0.75 | KNN |
| 0.65 | 0.676 | 0.172 | 1 | 0.294 | 1 | KNN |
| 0.7 | 0.635 | 0.172 | 1 | 0.294 | 1 | KNN |
| 0.75 | 0.635 | 0.172 | 1 | 0.294 | 1 | KNN |
| 0.8 | 0.635 | 0.069 | 1 | 0.129 | 1 | KNN |
| 0.85 | 0.635 | 0.069 | 1 | 0.129 | 1 | KNN |
| 0.1 | 0.459 | 0.828 | 0.222 | 0.545 | 0.407 | XGBoost |
| 0.15 | 0.514 | 0.759 | 0.356 | 0.55 | 0.431 | XGBoost |
| 0.2 | 0.581 | 0.621 | 0.556 | 0.537 | 0.474 | XGBoost |
| 0.25 | 0.608 | 0.621 | 0.6 | 0.554 | 0.5 | XGBoost |
| 0.3 | 0.622 | 0.621 | 0.622 | 0.562 | 0.514 | XGBoost |
| 0.35 | 0.662 | 0.621 | 0.689 | 0.59 | 0.562 | XGBoost |
| 0.4 | 0.662 | 0.552 | 0.733 | 0.561 | 0.571 | XGBoost |
| 0.45 | 0.676 | 0.552 | 0.756 | 0.571 | 0.593 | XGBoost |
| 0.5 | 0.662 | 0.517 | 0.756 | 0.545 | 0.577 | XGBoost |
| 0.55 | 0.662 | 0.517 | 0.756 | 0.545 | 0.577 | XGBoost |
| 0.6 | 0.676 | 0.448 | 0.822 | 0.52 | 0.619 | XGBoost |
| 0.65 | 0.662 | 0.414 | 0.822 | 0.49 | 0.6 | XGBoost |
| 0.7 | 0.649 | 0.379 | 0.822 | 0.458 | 0.579 | XGBoost |
| 0.75 | 0.649 | 0.379 | 0.822 | 0.458 | 0.579 | XGBoost |
| 0.8 | 0.676 | 0.345 | 0.889 | 0.455 | 0.667 | XGBoost |
| 0.85 | 0.662 | 0.31 | 0.889 | 0.419 | 0.643 | XGBoost |
| 0.1 | 0.446 | 0.862 | 0.178 | 0.549 | 0.403 | LightGBM |
| 0.15 | 0.446 | 0.759 | 0.244 | 0.518 | 0.393 | LightGBM |
| 0.2 | 0.514 | 0.724 | 0.378 | 0.538 | 0.429 | LightGBM |
| 0.25 | 0.527 | 0.724 | 0.4 | 0.545 | 0.438 | LightGBM |
| 0.3 | 0.554 | 0.69 | 0.467 | 0.548 | 0.455 | LightGBM |
| 0.35 | 0.568 | 0.655 | 0.511 | 0.543 | 0.463 | LightGBM |
| 0.4 | 0.676 | 0.655 | 0.689 | 0.613 | 0.576 | LightGBM |
| 0.45 | 0.649 | 0.586 | 0.689 | 0.567 | 0.548 | LightGBM |
| 0.5 | 0.649 | 0.552 | 0.711 | 0.552 | 0.552 | LightGBM |
| 0.55 | 0.662 | 0.517 | 0.756 | 0.545 | 0.577 | LightGBM |
| 0.6 | 0.649 | 0.483 | 0.756 | 0.519 | 0.56 | LightGBM |
| 0.65 | 0.676 | 0.483 | 0.8 | 0.538 | 0.609 | LightGBM |
| 0.7 | 0.662 | 0.448 | 0.8 | 0.51 | 0.591 | LightGBM |
| 0.75 | 0.716 | 0.414 | 0.911 | 0.533 | 0.75 | LightGBM |
| 0.8 | 0.703 | 0.345 | 0.933 | 0.476 | 0.769 | LightGBM |
| 0.85 | 0.703 | 0.345 | 0.933 | 0.476 | 0.769 | LightGBM |
| 0.1 | 0.392 | 1 | 0 | 0.563 | 0.392 | Stacking |
| 0.15 | 0.392 | 1 | 0 | 0.563 | 0.392 | Stacking |
| 0.2 | 0.392 | 1 | 0 | 0.563 | 0.392 | Stacking |
| 0.25 | 0.432 | 0.966 | 0.089 | 0.571 | 0.406 | Stacking |
| 0.3 | 0.486 | 0.897 | 0.222 | 0.578 | 0.426 | Stacking |
| 0.35 | 0.527 | 0.759 | 0.378 | 0.557 | 0.44 | Stacking |
| 0.4 | 0.608 | 0.69 | 0.556 | 0.58 | 0.5 | Stacking |
| 0.45 | 0.649 | 0.621 | 0.667 | 0.581 | 0.545 | Stacking |
| 0.5 | 0.662 | 0.517 | 0.756 | 0.545 | 0.577 | Stacking |
| 0.55 | 0.662 | 0.483 | 0.778 | 0.528 | 0.583 | Stacking |
| 0.6 | 0.757 | 0.483 | 0.933 | 0.609 | 0.824 | Stacking |
| 0.65 | 0.716 | 0.379 | 0.933 | 0.512 | 0.786 | Stacking |
| 0.7 | 0.716 | 0.345 | 0.956 | 0.488 | 0.833 | Stacking |
| 0.75 | 0.676 | 0.207 | 0.978 | 0.333 | 0.857 | Stacking |
| 0.8 | 0.689 | 0.207 | 1 | 0.343 | 0.857 | Stacking |
| 0.85 | 0.608 | 0 | 1 | 0 | 0 | Stacking |

Model performance (accuracy, sensitivity, specificity, F1 score, and precision) is calculated across thresholds ranging from 0.10 to 0.60. Threshold optimization was performed exclusively using the validation dataset to avoid information leakage from the test set. The table illustrates how different thresholds influence predictive balance across models, particularly in the Random Forest model.
